# Peptidic Connexin43 Therapeutics in Cardiac Reparative Medicine

**DOI:** 10.3390/jcdd8050052

**Published:** 2021-05-05

**Authors:** Spencer R. Marsh, Zachary J. Williams, Kevin J. Pridham, Robert G. Gourdie

**Affiliations:** 1 Fralin Biomedical Research Institute at VTC, Virginia Tech, Roanoke, VA 24016, USA; srmarsh@vt.edu (S.R.M.); zacharyjw@vt.edu (Z.J.W.); kjprid89@vt.edu (K.J.P.); 2 Center for Heart and Reparative Medicine Research, Virginia Tech, Roanoke, VA 24016, USA; 3 Translational Biology Medicine and Health Graduate Program, Virginia Tech, Roanoke, VA 24016, USA; 4 Department of Biomedical Engineering and Mechanics, Virginia Tech, Blacksburg, VA 24061, USA; 5 Department of Emergency Medicine, Virginia Tech Carilion School of Medicine, Virginia Tech, Roanoke, VA 24016, USA

**Keywords:** connexin43, peptide, cardiac disease, cardiac therapeutic, drug delivery

## Abstract

Connexin (Cx43)-formed channels have been linked to cardiac arrhythmias and diseases of the heart associated with myocardial tissue loss and fibrosis. These pathologies include ischemic heart disease, ischemia-reperfusion injury, heart failure, hypertrophic cardiomyopathy, arrhythmogenic right ventricular cardiomyopathy, and Duchenne muscular dystrophy. A number of Cx43 mimetic peptides have been reported as therapeutic candidates for targeting disease processes linked to Cx43, including some that have advanced to clinical testing in humans. These peptides include Cx43 sequences based on the extracellular loop domains (e.g., Gap26, Gap 27, and Peptide5), cytoplasmic-loop domain (Gap19 and L2), and cytoplasmic carboxyl-terminal domain (e.g., JM2, Cx43tat, CycliCX, and the alphaCT family of peptides) of this transmembrane protein. Additionally, RYYN peptides binding to the Cx43 carboxyl-terminus have been described. In this review, we survey preclinical and clinical data available on short mimetic peptides based on, or directly targeting, Cx43, with focus on their potential for treating heart disease. We also discuss problems that have caused reluctance within the pharmaceutical industry to translate peptidic therapeutics to the clinic, even when supporting preclinical data is strong. These issues include those associated with the administration, stability in vivo, and tissue penetration of peptide-based therapeutics. Finally, we discuss novel drug delivery technologies including nanoparticles, exosomes, and other nanovesicular carriers that could transform the clinical and commercial viability of Cx43-targeting peptides in treatment of heart disease, stroke, cancer, and other indications requiring oral or parenteral administration. Some of these newly emerging approaches to drug delivery may provide a path to overcoming pitfalls associated with the drugging of peptide therapeutics.

## 1. Connexin43-Formed Channels

The subunit of gap junction channels, connexin proteins, are widely expressed in the heart, as well as throughout the body [1,2,3,4]. Connexins are crucially important to cardiac electrophysiology, having direct and indirect assignments in facilitating the propagation of action potentials between cardiomyocytes [5,6]. Connexin43 (Cx43 gene name *GJA1*) is the main cardiac connexin, being largely expressed by cardiomyocytes, but also expressed by fibroblasts, myofibroblasts, and vascular cells in the heart [7,8,9,10]. The half-life of Cx43 is ~1.5 h, which is approximately 1000 times shorter than that of cardiac collagen—a prodigious rate of turnover hinting at the substantial and still not as yet fully understood functions of Cx43 [6,11,12]. As is the case with all 21 expressed connexins that are encoded by the human genome [13], Cx43 is a transmembrane protein, with four membrane-spanning domains and cytoplasmically located loop, amino- and carboxyl-terminal domains [14].

**Figure 1:**
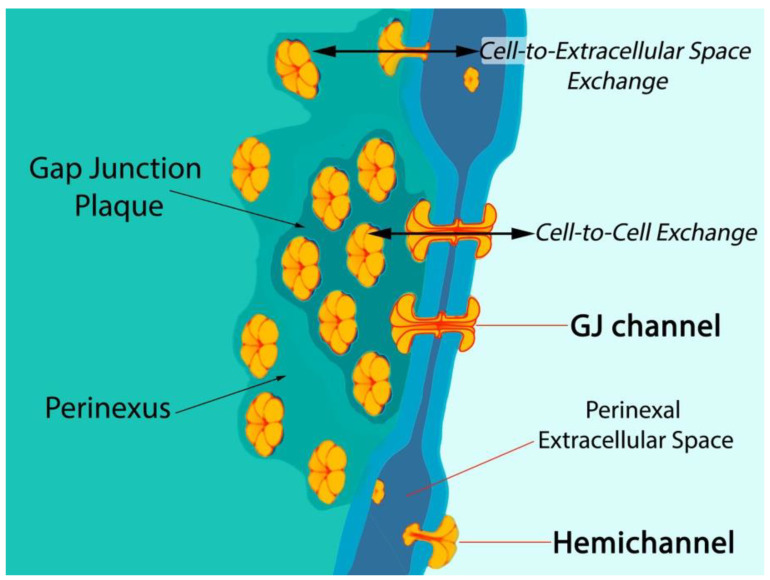
Model of gap junction between two cells. Connexon hemichannels dock to form intercellular channels coupling the cells, enabling cytoplasmic exchange of ions and small molecules typically less than 1000 Da in molecular weight. Undocked hemichannels, concentrated in the perinexus surrounding the gap junction [8], are typically closed but can open in response to prompts like ischemic stress. Open hemichannels thereby underpin flow of channel-permeant molecules from the cytoplasm into the extracellular space.

Six connexin subunits oligomerize during trafficking to the cell membrane to form a connexon channel, which when open is capable of transferring ions and other small molecules (typically < 1000 daltons) in a gated and relatively nonselective manner (Figure 1) [5,6]. Connexons formed by most connexins can occur in two states in the cell membrane, either as undocked hemichannels (HCs) or docked with another connexon from an adjacent cell to form a gap junction (GJ) channel. Consequently, connexons can perform two distinct information exchange functions: first, as undocked connexon HCs, which enable two-way exchange between the cell interior and the extracellular milieu; and second, as docked connexons in GJ channels providing a regulated pathway for cytoplasmic exchange between cells without recourse to the extracellular space. Undocked HCs in the cell membrane comprised of Cx43 are typically closed but can open in response to various prompts including ischemic injury [15,16,17,18,19,20,21,22].

The biology of connexin structure and function and particularly that of Cx43 and its functions in intercellular communication have been covered extensively by us and others [6,7,9,10,23,24]. A focus of this review is the biology and pathophysiology of HCs formed by Cx43 in the myocardium, as well as the growing literature on the opportunities and barriers to pharmacological targeting of these channels in the treatment of heart disease. There is a wealth of preclinical data indicating the potential for therapeutic benefit from targeting HC activity by drugs mimicking the sequence of Cx43 in experimental models of human pathology, including those involving injury to the skin, heart, and brain [4,24,25,26,27,28]. If translation of these Cx43-targeting drugs to the clinic is to occur, careful attention to addressing questions of how to safely and effectively deliver drugs based on short peptides is required.

## 2. Connexin43 and Myocardial Pathology

Numerous cardiac disease processes and pathologic mechanisms have been linked to Cx43 (recent reviews of this topic include [9,24,28,29,30,31]). Of these, the most studied and clinically significant are myocardial infarction (MI) and ischemia reperfusion (I/R) injuries of the heart linked to this syndrome [6]. In the post-MI environment, Cx43 expression is reduced and localization of Cx43 is disturbed (lateralized) in myocytes at the infarct border from as soon as 1 h following ischemic insult [32]. Long-term Cx43 downregulation and lateralization are thought to contribute to the increased potential for electrical conduction disturbance in hearts subject to ischemic injury, particularly re-entrant arrhythmias triggered in tissues surrounding a MI—the infarct border zone (IBZ) [33,34].

Among the post-translational changes that appear to be significant to the injury status of the heart are alterations in the phospho-status of Cx43. Ek-Vitorin and colleagues found that Cx43 phosphorylated at a consensus PKC (protein kinase C) site, serine 368, was retained at intercalated disks during early ischemia and that this retention was associated with cardioprotection [35]. Pertinently, phosphorylation of Cx43 at S368 (Cx43 pS368) is correlated with reduced activity of Cx43-formed HCs [35,36,37]. Phosphorylation of Cx43 associated with mitochondrial membranes has also been linked to I/R injury [38]. Phosphorylation at S368 and certain other serines in CT (e.g., Serines 262 and 373) are dependent upon dephosphorylation of a serine 365, “gatekeeper” amino acid [39]. The downstream changes to Cx43 that ensue following S365 dephosphorylation result in changes to the gating and perm-selectivity of Cx43-formed channels, as well as the dysregulation of Cx43-ZO-1 interactions [40]. Another post-translational modification significantly affecting Cx43 is ubiquitination [41]. One of first pieces of evidence that Cx43 is ubiquitinated was provided by Laing and Beyer [42]. Cx43 ubiquination acts as a signal for gap junction endocytosis by recruiting the ubiquitin binding protein Eps15 (epidermal growth factor receptor substrate-15) [43]. Significantly, Cx43 ubiquitination has been proposed to control its post-endocytic sorting from early endosomes to lysosomes, controlling degradation of Cx43 [44]. Ubiquitinated forms of Cx43 are recognized by the endosomal sorting complex required for transport (ESCRT) sorting system, which is responsible for sorting into endosomes and exosomes.

Post-translational modifications to Cx43 appear to be of particular significance at the edge of GJs—in the perinexus—a specialized nanodomain where Cx43 HCs are concentrated (Figure 1) [6,45]. During the acute phase of an MI, large-scale opening of Cx43 HCs occurs [18,46]. HC opening may be also exacerbated during the reperfusion phase of an I/R injury. Pro-inflammatory and injury-spread signals resulting from unregulated opening of Cx43 HCs are likely crucial to the generation and severity of I/R damage [6,10,47,48]. The flux of cytoplasmic contents released by HCs is thought to contribute to a “bystander effect”, wherein cell loss and necrotic damage are caused in otherwise healthy tissue adjacent to the primary site of injury. Intercellular coupling by GJs may also contribute to injury spread via the bystander effect [49,50,51]. Examples of tissues susceptible to the “bystander effect” include myocardium-at-risk [52], cardiac muscle tissue surrounding the ischemic core of an MI. Adenosine triphosphate (ATP) is thought to be one of the more consequential pro-inflammatory molecules released by HCs [17,53,54,55]. Extracellular gradients of ATP facilitate directed migration by neutrophils to the sites of injury, disease, or infection [56]. This purinergic signal also induces production of extracellular nets—a lethal behavior elicited by inflammatory cells that likely contributes to myocyte death and the extent of myocardium-at-risk following MI [57].

Arrhythmogenic right ventricular cardiomyopathy (ARVC) is a pathology that is likely directly impacted by Cx43 expression and phosphorylation; ARVC patients experience a loss of desmosomes, a specialized cell–cell junction [58,59,60]. Among key proteins in desmosomes is desmoplakin—the gene-encoding desmoplakin was the first desmosomal gene to be linked to ARVC [61]. When this protein is deleted from cardiomyocytes, the cells exhibit large reductions in GJs and Cx43, as well as changes to Cx43 phospho-status, associated with loss of intercellular communication [62]. A mutation of another desmosomal protein, plakophilin-2 (PKP2) has shed further light on ARVC disease mechanisms [60]. Cardiomyocyte-specific knockout of PKP2 in mice results in generation of an arrhythmogenic substrate (Ca^2+^ dysregulation) that was apparent at time points before overt structural changes in myocardium associated with ARVC-like disease (e.g., fibrosis). Interestingly, Cx43 ablation relieved these functional deficits in the ventricle. Moreover, treatment with a selective peptide-based blocker of Cx43 HCs normalized arrhythmogenic Ca^2+^ dynamics—suggestive of a role for HCs in the formative stages of ARVC pathogenesis.

Duchenne muscular dystrophy (DMD) is an X-linked recessive disorder resulting from loss-of-function of dystrophin [63]. In skeletal and cardiac myocytes cells, dystrophin is a membrane-associated protein that links the cytoskeleton and the surrounding extracellular matrix, thereby providing mechanical integrity during muscle cell work [64]. The absence of dystrophin triggers a cascade of biochemical changes that ultimately leads to the death of muscle cells and fibrosis associated with myofibroblast proliferation. The primary culprit in muscle loss appears to be intracellular calcium overload and the triggering of oxidative stress pathways leading to cell death. This chain of events results in over 50% of DMD patients experiencing cardiomyopathy by the age of 10, with over 90% of patients experiencing cardiac dysfunction by the age of 18 [65]. The particular danger in DMD in relation to Cx43 lies in progression to dystrophic cardiomyopathy, a pathology strongly affecting Cx43 expression and function [66]. Dystrophic cardiomyopathy patients experience Cx43 remodeling away from intercalated discs to lateralized positions, increased Cx43 expression, and a propensity to develop disturbances to cardiac conduction. Recent evidence suggests that unregulated opening of HCs is central to the role of Cx43 in the pathogenesis of DMD [67,68,69]. S-nitrosylation and reduced phosphorylation of Cx43 at serines S325/S328/S330 have been reported to be associated with HC activation, arrhythmogenesis, and ventricular remodeling in the Mdx mouse model of DMD [67]. A recent study has suggested that inhibition of aberrant HC activity in skeletal muscle macrophages neighboring Cx43 nonexpressing fibers in symptomatic DMD mice leads to prevention of Cx43 remodeling in the heart and protection from the loss of skeletal and cardiac muscle cells that characterize this disease [70].

Yet another example of probable involvement of Cx43 HCs in cardiac disease processes comes from a study of myocytes expressing altered nuclear lamin A/C proteins, mutations associated with laminopathy—a disease in humans leading to heart failure and arrhythmias [71]. Cultured neonatal rat cardiomyocytes expressing pathologic human lamin A/C mutations exhibit altered microtubule structure, hemichannel localization and beating force, frequency, and contractile amplitude. The common endpoints of diseases such as ARVC, DMD, and laminopathy are muscle cell death and irreversible replacement of lost myocardium with scar tissue. The mounting evidence suggests that HC activation is a precursor to this fibrotic replacement, often in association with oxidative stress, dysregulated intracellular Ca^2+^ dynamics, disturbances to cardiac conduction, and arrhythmias.

## 3. Cx43-Targeting Therapeutic Peptides

**Figure 2:**
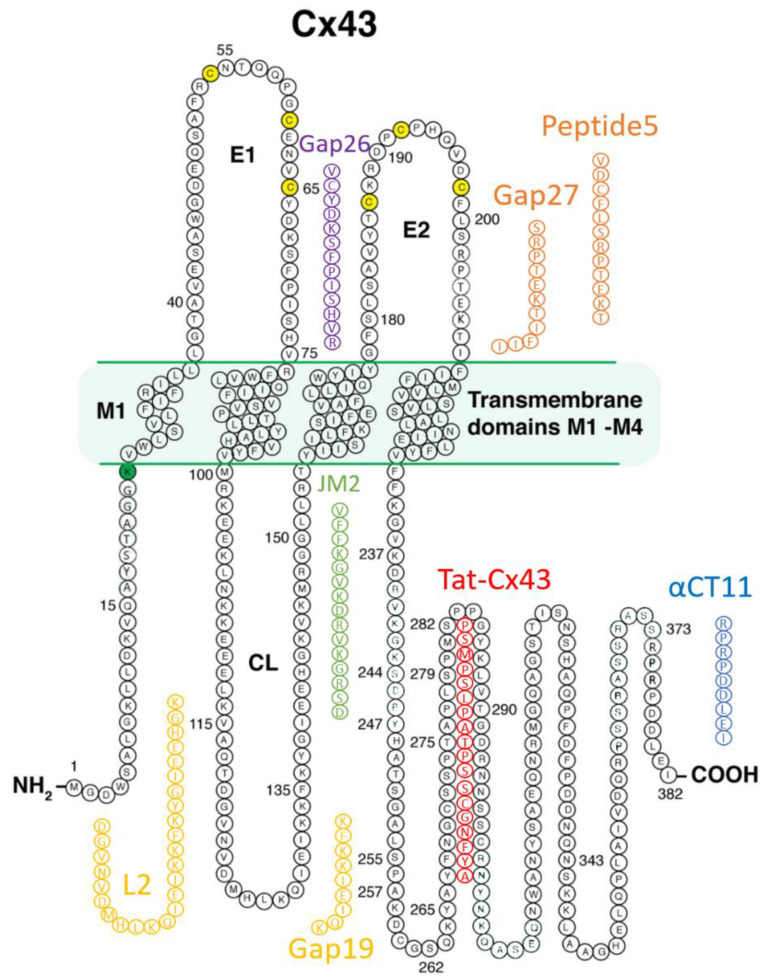
Connexin-43 mimetic peptides discussed in this review.

Prior to the new millennium, connexins were not considered well suited for pharmacological targeting for reasons that included their minimal extracellular profile and, thus, absence of an obvious external receptor for ligand binding. However, over the last decade, there has been growing interest in the potential for connexin pharmacology. This has occurred as a result of growing evidence of roles for connexin-formed channels in clinically relevant phenomena including arrhythmias, cancer, wound healing, inflammation, and tissue injury responses, as well as the growing understanding of the potential contribution of HCs and GJs to the “bystander effect”, such as what occurs in myocardium-at-risk following MI. Additionally, a path to drugging Cx43 has been illuminated by the development of peptides that mimic or bind to Cx43 [4,6,26,72,73,74,75,76,77,78,79,80,81,82,83,84,85,86,87,88,89,90,91,92,93,94]. Figure 2 provides a summary of these peptidic therapeutic candidates, and Table 1 shows results to date pertinent to heart disease as reported in preclinical studies, each of which is discussed in the following sections.

### 3.1. Mimetic Peptides Based on Cx43 Extracellular Domains

#### 3.1.1. Gap26 and Gap27

The first connexin mimetic peptides discovered to have biological activity were Gap26 and Gap27, which contain segments of extracellular loops (EL) 1 and 2, of Cx43, respectively [86,87,88,89,90,91,92]. In initial reports, these peptides were found to inhibit the synchrony of contractions between pairs of cell aggregates prepared from embryonic chick hearts [89]. These results led the authors to propose that Gap26 and Gap27 may be inhibiting gap junctional coupling. The mechanisms by which Gap26/27 interfere with connexin-formed channels remain to be fully understood. A review characterizing the possible mechanism of action for extracellular loop peptides is found in the literature by Berthoud et al. [90]. It is suggested that these peptides may reversibly interact with the EL domain of HCs, thereby preventing the interaction and docking of HCs to form GJ channels [90,91]. Evidence supporting this hypothesis comes from atomic force microscopy experiments in which Gap26 was covalently bonded to the scanning tip and exhibited binding interactions with Cx43 EL domains [92]. The data indicate that peptides derived from the second Cx43 EL (EL2) may interact with EL2 and not EL1, while EL1 peptides synergistically increase inhibition of Cx43 channel formation when co-administered with an EL2-based peptide [93]. Peptide mimetics of connexin extracellular loop domains have also been reported to inhibit the activity of channels formed by other connexins, including Cx40 and Cx37 [94,95,96,97,98].

Another possibility for the effects of Gap26/27 mode of action may be direct interactions of peptides with existing GJs, resulting in separation of docked channels or in blocking of GJ gating. While binding with existing GJs is possible in principle and has been proposed, it has yet to be proven. A theory that has gained more traction is direct interaction of Gap26/27 with HCs, which is presumed to block the formation of GJs. The most robust evidence for this proposal comes from the short time course in which EL peptides block HC activity, relative to GJs [88]. HCs are inhibited within a few minutes, whereas GJ inhibition occurs over time scales of an hour or more. A recent advance in both the administration and mechanism of EL peptides has been the development of a lipidated version of a Gap27-like peptide (SRPTEKT-Hdc) by Burt and colleagues [99]. This chemical modification increased the potency of EL-peptide with respect to effects on Ca^2+^-wave propagation, dye coupling, and HC-mediated dye uptake in cultured cells, wherein it showed activity at nanomolar concentrations, rather than in the micromolar range, as occurred for nonlipidated peptide. Interestingly, SRPTEKT-Hdc did not have similar effects on cells expressing Cx43 with mutations at S368, suggesting that phosphorylation at this locus may be involved in the mode of action of Gap26/27-like peptides [99].

Some of the earliest studies on Cx43 EL mimetic peptide effects in heart cells were carried out in vitro on monolayers of cultured neonatal rat ventricular myocytes subject to simulated ischemia (oxygen-glucose deprivation)—a challenge that results in upregulated HC opening within an hour [46]. Treatment of myocyte monolayers that were subject to simulated ischemic injury with Gap26 reduced HC opening and improved myocyte viability up to 80% of control levels. Gap26 and 27 have also demonstrated cardioprotective effects in intact hearts in ischemia reperfusion (IR) injury models in rodents [75,76,77,78]. Hawat and coworkers found that single bolus injections of Gap26 or Gap27 at 1 μg/kg into the jugular vein of rats subjected to MI in vivo, either prior to or following the ischemic episode, caused infarct size reductions in injured ventricles of up to 61% relative to control rats [77]. In addition to acute treatments, there is also evidence that repeated dosing of EL-based peptides can have beneficial effects in chronic models of heart disease. Lucero et al. [79] administered Gap27 continuously via osmotic minipumps in a high-output heart failure model in adult male rats for 4 weeks. This recurring and sustained treatment regime resulted in significant improvements in heart mechanical function, lowered arrhythmia burden and reduced cardiac hypertrophy, as compared to control hearts—supporting further investigations of the therapeutic use of EL-based peptides in mitigating progression to heart failure.

#### 3.1.2. Peptide5

Peptide5, developed by Green and coworkers, is another well-studied Cx43 EL mimetic, incorporating the SRPTEK domain found within Gap27, with the full sequence VDCFLSRPTEKT [100]. This peptide was developed by screening various sequences from Cx43 EL domains for optimized potential to inhibit neuronal death in an in vitro model of spinal cord injury. Peptide5 inhibits HCs at a concentration of 5 uM, while concentrations of two orders of magnitude greater are required for GJ inhibition. Follow-up work has demonstrated inflammatory dampening effects, as well as neuroprotective capabilities [101,102,103]. This was supported by research which utilized Peptide5 in L3-L5 lumbar spinal injury abatement; administration of Peptide5 reduced NOD-like receptor protein 3 (NLRP3) inflammasome complex, a key mediator of neuroinflammation [104]. Ongoing studies have indicated that Cx43 HC activity has a direct role in inflammation and inflammasome activation, while Peptide5 blocks the development of these inflammatory signals [105]. Recent work by Kim and coworkers determined that the SRPTEKT region is likely the operational stretch of Peptide5 with respect to HC inhibition [93]. Other investigations of Peptide5 in preclinical models have included elucidation of its salutary effects on retinal pigment epithelial cell barrier function [106] and chronic kidney disease [107]. To date, there are no publications on Peptide5 efficacy in preclinical models of heart disease.

### 3.2. Mimetic Peptides Based on Cx43 Cytoplasmic Loop Domains

#### Gap19 and L2

Gap19 is a peptide mimicking a nine amino acid sequence (KQIEIKKFK) within the cytoplasmic CL domain of Cx43 [20,80]. The Gap19 sequence is located within a sub-region of the CL referred to as L2, which has been shown to be involved in Cx43 self-interactions with domains on the CT of Cx43, with the balance of data suggesting the molecular mechanism of Gap19 involves inhibition of Cx43 CL-CT interactions. This mode-of-action is further supported by studies of the effects of Gap19 on Ca^2+^ sensitive gating of Cx43 CT truncation mutants [108]. Gap19 also seems likely to modify interaction with interacting partners such as ZO-1, whose PDZ2 also has strong affinity for the portion of the Cx43 CT that interacts with Gap19 [85]. Importantly, Gap19 appears to show selectivity for Cx43s HCs, only affecting GJ activity at high concentrations [20,109]. Recent studies have also indicated that Gap19 may have a bimodal effect on gating, decreasing gating of a 210 pS conductance state but also significantly increasing the open probability of an 80 pS substate of HCs formed by Cx43 [110].

The first demonstration that Gap19 may have effects in a preclinical model of cardiac disease was an investigation of its inhibitory actions on metabolic inhibition-enhanced HC opening, protecting myocytes against volume overload and cell death following IR injury in vitro, as well as modestly decreasing MI size after myocardial IR injury to mouse hearts [80]. There is evidence the cardioprotective effects of Gap19 may relate to inhibition of mitochondrial Cx43 [81]. Treatment of mitochondria isolated from subsarcolemmal zones of ventricular myocytes with Gap19 caused reductions in the velocity of potassium influx– a significant aspect of oxygen consumption in these organelles. The facility of Gap19 to act as a modifier of HC gating also provided a tool in studies of the role of Cx43 HCs in the pathogenesis of ARVC and DMD, wherein Gap19 was found to have anti-arrhythmic effects in mouse models of these diseases [60,82].

As Gap19 targets cytoplasmic domains of Cx43 the peptide must gain entry to cells for activity—an ability that was achieved in many past studies via conjugation to TAT cell penetration peptide [80,111]. In a recent innovation, Rupenthal et al. linked Gap19 to the cell-penetrating peptide Xentry (XG19), determining that the novel peptide exhibited increased levels of cellular uptake [112]. The authors also reported preferential uptake of XG19 at sites of hypoxic injury, leading to increased delivery to injured cells. Given the cardioprotective effects of Gap19 [80], as well as reports that this peptide decreases oxidative stress, inflammatory cytokine levels, and senescence in endothelial cells [113], the facility of Gap19 to selectively target hypoxic cells and abrogate ischemia-related pathology supports the case for ongoing research on this peptide as a candidate therapeutic.

### 3.3. Mimetic Peptides Based on Cx43 Cytoplasmic Terminal Domain

#### 3.3.1. alphaCT/αCT Peptides

The first peptide incorporating a sequence from the cytoplasmic CT of Cx43 demonstrating biological activity was reported by the Gourdie lab in 2005 [85]. The peptide, known as alphaCT1 (αCT1), includes an antennapedia cell penetration sequence and the CT-most nine amino acids of Cx43, mimicking its ZO-1 PDZ2 binding ligand. In cell culture models, αCT1 was shown to increase GJ size and coupling, as well as reduce levels of HC activity [85,114]. The peptide was later found to also bind domains within Cx43 itself [83,115], including the CT H2 domain [116]. At present, it is unclear whether these interactions occur within the same Cx43 molecule or involve dimeric CT-CT interactions between different Cx43 molecules in the same connexon. The interaction of αCT1 with the H2 domain is associated with the increase in a PKCe-mediated phosphorylation of Cx43 at S368 [83,117] a post-translational modification that has been linked to reduced activity of Cx43-formed channels [36,37,39].

Whilst initially developed as a research tool, αCT1 was subsequently found to increase closure rate and reduce inflammation and scarring in skin and corneal wound healing studies in rodents [4,26,118,119]. Based on such results, Ghatnekar and colleagues at FirstString Research Inc. have advanced the peptide to testing in humans, reporting outcomes from Phase II clinical trials on the safe and efficacious use of αCT1 in promoting faster closure of venous leg ulcers and diabetic foot ulcers, as well as in surgical scar mitigation [120,121,122]. Preclinical studies of αCT1 also indicate potential for its use in treatment of heart disease. αCT1 has been shown to have anti-arrhythmic effects [117], as well as demonstrating cardioprotective properties in mouse hearts subject to ischemia reperfusion injuries [83]. A short version of αCT1 25 mer called αCT11 (nine amino acids) was found to be significantly more potent in reducing ischemia reperfusion injury in Langendorff-perfused mouse hearts than αCT1 [83]. Moreover, when αCT11 was administered postmyocardial infarction in vivo, it reduced infarct size by nearly 50%, raising the prospect that this short peptide could have potential for clinical translation [84].

Of further interest with respect to the activity and mechanism of Cx43 CT peptide mimetics is that Shaw and coworkers recently demonstrated that exogenous overexpression of a naturally occurring 20 kDa isoform of Cx43 corresponding to its CT domain (GJA1-20k) reduces myocardial infarct size in mouse hearts subject to ischemia reperfusion injury [123]. The effects reported appear to be similar to the cardioprotective activity observed for αCT1 and αCT11 [83,84]. GJA1-20k comprises considerably more of the Cx43 CT sequence than αCT11 and αCT11 and thus care must be taken to avoid over extending interpretation beyond available data. Nonetheless, it may be material to the cardioprotective mechanism of CT mimetics that the GJA1-20k Cx43 isoform associates with the outer mitochondrial membrane and affects mitochondrial motility and biogenesis [123].

#### 3.3.2. CT9

In 2010, Leybaert and colleagues published studies of peptides containing sequences identical to αCT1, including a molecule called CT9 based on the CT-most nine amino acids of Cx43 (RPRPDDLEI) [124]. This group further reported that Cx43 CT-based peptides promoted thrombin-induced Cx43 HC opening. A more recent study showed the potential for CT9 to acutely activate HCs in the presence of TNF alpha or low extracellular Ca^2+^ concentrations—though CT9 had no effect on HCs in the absence of lowered [Ca^2+^] or TNFα [125]. These data raise a paradox, as the results from αCT1 and αCT11 suggest that under some circumstances the effects of these peptides are consistent with reduced HC activity—including reductions in HC density within the perinexus and HC permeation [114], together with increased S368 phosphorylation of Cx43 [83,117,126]. The context of measurement and other experimental details may be considerations in addressing these differing results. First, it is worth noting that generally CT9 has not been shown to promote HC opening in its own right—experimental accounts indicate that CT9-induced HC activation typically occurs in the presence of cofactors, e.g., a HC-opening treatment such as lowered extracellular [Ca^2+^] [124,125]. In an interesting parallel, Palatinus et al. found that αCT1-enhancement of PKC-mediated phosphorylation of the Cx43 was dependent on peptide treatment being a cofactor in accompanying a cellular injury [126]. Second, descriptions of the direct HC-activating effects of CT9 have thus far come from studies of isolated cells. Given the perinexal nanodomain is not present, or is disrupted in unitary cells, it may be that regulatory elements normally at-hand in this key HC niche are not available to exert modulatory effects on HC activity. Third, and probably most important of all, reports of the time course of activation of HCs by CT9 have thus far been confined to recording of less than a few seconds [125]. The HC-opening effects of CT9 over time intervals extending for minutes or hours are presently unknown. The induction of channel inhibitory phosphorylations of Cx43 or changes to GJ/HC organization reported following αCT1/αCT11 treatment were measured over time intervals considerably longer than a few seconds, i.e., many minutes to multiple hours and days [83,84,114,117]. Thus, one path to reconciling differing interpretations on CT9 vs. αCT1/αCT11 could be that the phenomena observed in response to isolated Cx43 CT fragments depend on the time frame of measurement—namely, that there may be a short-term HC-activating effect and longer-term HC downregulation. A pertinent consideration here may be the salutary effect of ischemic preconditioning by short bursts of hypoxia. Acute ischemia is a known HC-opening prompt [46], but longer-term preconditioning results in reduced activity of Cx43-formed channels and enhanced cardioprotection from ischemia reperfusion injury [48]. Thus, the apparently differing results on CT9 and αCT1/αCT11 provide a fruitful dilemma. Ongoing research of the paradoxes inherent in the findings from different groups may lead to new approach to treatment of diseases of the heart.

#### 3.3.3. Other Cx43 CT Mimetic or Targeting Peptides

A number of other peptides based on or directly binding to the Cx43 CT have been reported. The JM2 peptide mimicking 15 amino acids located in the juxtamembrane region of Cx43 CT (VFFKGVKDRVKGRSD) has been shown to inhibit ATP release triggered by low extracellular Ca^2+^ concentrations in cultures of human microvascular endothelial cells [127]—as such, JM2 appears to show Cx43 HC inhibitory activity. The JM2 peptide incorporates a sequence of Cx43 thought to function as a microtubule binding domain [128]. JM2 has demonstrated utility in preclinical studies as an anti-inflammatory agent in the foreign body response [127] and in the targeted killing of glioblastoma stem cells [129]. Interestingly, another peptide based on sequence adjacent to juxtamembrane-located JM2 on the Cx43 CT, Tat-Cx43 266-283 has also shown efficacy in in vitro and in vivo models in decreasing glioblastoma stem cell viability [130].

Delmar and coworkers used biophysical techniques to identify novel peptide sequences that bind to the CT of Cx43 [131]. One molecule isolated via this method named RXP-E was found to prevent acidification-induced GJ uncoupling and preserve action potential propagation in cultured myocytes, suggesting potential as an anti-arrhythmic [132]. Whilst neither mimicking nor known to interact with the Cx43 CT, AAP10 is a short peptide characterized by affecting Cx43 structure and function, including modulating Cx43 phosphorylation. AAP10 and a group of related molecules have shown preclinical potential in treating cardiac disease [133]. This group includes Rotigaptide (ZP123) and Danegaptide (ZP1609), which were developed in an effort to chemically engineer more stable and potent variants of the parent AAP10 molecule [133]. The mode-of-action of these peptide-based molecules remains to be characterized, though AAP10 has been shown to increase intercellular communication between myocytes [134]. Studies in porcine and canine models have demonstrated that Rotigaptide has anti-arrhythmic properties [135,136,137]. Work in pigs have also determined that when Danegaptide was given prior to an IR injury it reduced MI severity to levels comparable to ischemic postconditioning [138]. Regrettably, accounts of the Phase II clinical trials on Danegaptide suggests that this drug candidate provides no significant cardioprotective effect in humans when administered acutely following ischemic injury [139]. Clinical studies of Rotigaptide have also been undertaken for indications including vascular disease, coronary artery disease, and heart rhythm, though these studies were terminated without follow-up reports in the primary literature [140].

## 4. Barriers to Clinical Translation of Cx43-Targeting Peptidic Therapeutics

There has been extensive research and development activity on peptidic therapeutics –recent reviews on the topic include [141,142]. Worldwide over 60 peptide drugs have passed regulatory approval for use in humans—with metabolic disorders and cancer being the most commonly targeted disease indications. Insulin was the first and probably longest-used therapy of this type [143,144], but the broad gamut now includes drugs such as liraglutide and glucagon-like peptide 1, which are being used for treatment of metabolic disorders, with annual sales currently exceeding two billion US dollars. No molecules based on or targeting Cx43 have as yet been approved for clinical use, though as discussed in preceding sections, some have advanced to late-stage clinical testing [4,26].

Whilst interest is burgeoning, it is recognized that significant challenges exist in clinical translation of peptidic therapeutics [145]. This includes the need to resolve difficulties associated with the administration of peptidic therapeutics, as well as a requirement to improve the stability, bioavailability, and tissue and cell penetration properties of these drug candidates. Issues such as these have likely tempered the enthusiasm of the pharmaceutical industry in pursuing development of peptide drugs, even when supporting preclinical data is strong. Short linear peptides such as those exemplified by Cx43 mimetics may present special challenges due to their relatively short life in fluids like blood—limiting the time window that such molecules achieve concentrations within the body sufficient for bioactivity. In one example, our own studies of the nine-mer αCT11 suggest that it is degraded within 30 min of incubation in mouse serum at 37 C. In this respect, it is also notable that much of the clinical development of alphaCT peptides have been as topical treatments for chronic skin wounds or injuries to cutaneous tissue [26] where degradative processes can be controlled to some extent by external application of the drug and controlled release from protective delivery vehicles. In the final section of the review, we survey strategies that have or have potential to mitigate drawbacks linked to peptidic drugs and how these lessons could be applied to Cx43-targeting therapeutics.

### 4.1. Stability of Peptidic Drugs In Vivo

The most common concern raised on peptide-based drugs are questions on their stability in vivo. Some naturally occurring peptides such as insulin have been designed by evolution to resist proteolysis and breakdown [146]. However, for novel and synthetic peptides such as most Cx43 mimetics this remains an issue. Various chemical strategies have been used to extend the half-life of peptides in vivo including addition of chemical groups to side chains and the NT and CT of the molecule, use of enantiomeric and other non-natural amino acids to assemble all or part of the sequence, and peptide stapling and cyclization modifications to increase stability of peptides [144,145,146]. For example, the Cx43-targeting peptide Danegaptide represents an enantiomeric form (i.e., composed of L- amino acids) of the naturally occurring peptide AAP10 [133]. However, modifying peptides chemically to achieve goals such as improving stability in vivo may not come without cost. For example, consideration as to whether such modifications increase toxicity, and in particular immunotoxicity, needs to be taken into account. Controlled release from a polymeric vehicle or capsule is another approach to the slow breakdown of peptides, as well as for sustaining effective systemic concentration of the drug, reducing the need for repeated dosing. This approach has been employed with αCT1, wherein the peptide has been encapsulated in alginate-poly-l-ornithine microcapsules (150 μm) and delivered in normal and diabetic corneal injury models in rabbits [147,148]. More recently, αCT1-containing nanoparticles (100 nm) were generated using double emulsion-solvent evaporation method from poly(lactic-co-glycolic acid) (PLGA). Encapsulation of αCT1 in PLGA nanoparticles enabled release of peptide over periods of three weeks in solution, characterized by an initial burst of approximately half of the encapsulated drug over the first three days, followed by sustained availability of active peptide over the remaining two and a half weeks [149].

### 4.2. Cell and Tissue Penetration of Peptidic Drugs

In general, peptide-based drugs have relatively poor penetration characteristics, restricting delivery to internal organs and tissues, as well as complicating administration. For example, oral administration is not an option for peptidic therapeutics, as most protein sequences, bar a few select di- and tri-peptides, are rapidly hydrolyzed following ingestion by digestive processes [150]. With a few important exceptions, peptides also do not efficiently cross tissue boundaries such as the blood–brain, blood eye, and dermal barriers. In a well-characterized example, peptides derived from the rabies virus glycoprotein have been shown to cross the blood–brain barrier and under study for their potential to deliver therapeutic cargos into brain tissues [151]. The most common employed strategy used to improve the delivery of Cx43-targeting peptides is the addition of a cell penetration sequence (CPP). A number of such constructs, including antennapedia, TAT, and XEntry, have already been discussed in this review. There are accounts that a TAT-conjugated version of Gap19 shows ability to penetrate into brain parenchyma from the circulation [109], though it remains unclear that this is a general property of TAT-conjugated Cx43 mimetic sequences. The principal rationale for addition of a CPP is to enable peptide sequences to cross cell membranes and engage cytoplasmic targets—and as such may not be a useful modification for extracellularly located connexin sequences such as Gap26 and Gap27. Nonetheless, it is notable that lipidation of EL-loop peptides increases potency by an order of magnitude or more [99]. There is also evidence that under some circumstances a CPP may not be required for cell penetration. Jiang et al. reported that αCT11, a nine amino acid peptide, was able to cross into the cytoplasm of myocytes with an efficiency similar to that of its CPP-containing parent peptide αCT1 [83]. At present, the mechanism for this is unclear, though as Cx43-formed channels have been shown to be permeated by linear peptides of up to 15 amino acids [152], open HCs thus may provide a route by which the αCT11 nine-mer is able to gain access to its cytoplasmic targets [83].

### 4.3. The Immune System and Peptidic Drugs

One aspect of peptide-based drugs that is not often well broached in literature is their potential interaction with the immune system. This consideration is significant in the bioavailability of peptides in vivo, particularly after a second or third administration, where a primed immune system may more efficiently clear the molecule. However, the safety implications of repeated exposure are also of interest. This concern may be further enhanced by strategies such as chemical modifications, use of non-natural amino acids, and peptide cyclization, which while improving stability in vivo, may augment the ability of the immune system to distinguish the molecule as nonself. CPPs such as TAT may also increase engagement by the immune system, promoting antigen uptake, processing, and presentation by antigen-presenting cells [153]. Unfortunately, there are few data from preclinical studies of Cx43-targeting peptides on this issue. There is some information from clinical studies on αCT1 in its Granexin formulation for topical use on skin wounds, wherein one Phase I and three Phase II clinical trials found no indication of antibodies against the peptide in patient sera [120,121,122]. The treatment regimens for chronic wounds (diabetic foot ulcers and venous leg ulcers) in these studies involved repeated weekly dosing with topically applied αCT1 over a 3 month study period, with the independent contract research organization carrying out the work finding no occurrences of severe adverse events, including no evidence of immune responses to the peptide drug. Such reports are encouraging, and the outcome in this case may point to the benefit of clinical testing strategies focused on external administration of a gel containing the peptidic drug to the skin, where exposure to the immune system may be reduced.

### 4.4. The Potential of Extracellular Vesicles as Vehicles for Mimetic Peptides

Topical application of Cx43-based peptides to skin has shown success in clinical testing. However, the short half-life, poor tissue penetration characteristics, and potential for interaction with the immune system of peptidic drugs remain drawbacks on extending their use to clinical indications within the body such as myocardial infarction. Fortunately, technologies are on the horizon that may enable some of these concerns to be surmounted. One avenue that our laboratory is putting emphasis on in ongoing work is the encapsulation of Cx43 therapeutic drug candidates such as αCT11 in exosomes. Mounting evidence suggests that this class of small extracellular vesicle may enable shielding and protection of fragile drug cargos during transport within body fluids [154,155]. The ability of exosomes to release their encapsulated cargo into the cytoplasm of target cells also obviates the need for CPP sequences. Exosomes demonstrate immune privilege and certain types of these nanovesicles are adept at crossing tissues boundaries, including the blood–brain-barrier [156,157,158,159,160]. Mammalian milk turns out to be a rich source of exosomes [161,162,163] opening up the possibility of production at industrial scale. Milk exosomes also show a unique propensity to be taken up from the gut and transferred to the circulation, a characteristic that may enable oral administration of peptide therapeutics [164]. Considerable work is still required, including development of cost-effective isolation techniques for producing pharmaceutical grade exosomes in large amounts, reliable approaches to drug loading, and extensive preclinical and clinical testing of these novel formulations.

## 5. Concluding Remarks

There is strong evidence from preclinical and clinical research that targeting gap junctional Cx43 may provide a path to amelioration of human pathologies, including diseases of the heart, the focus of this review. The last two decades have seen the emergence of drug-like peptidic molecules mimicking or interacting with Cx43 that have shown therapeutic potential. A number of questions and opportunities are presented for ongoing study. First, the potent bioactivity of synthetic Cx43 fragments presents the interesting question of whether these molecules are amplifying a naturally occurring injury response mechanism. It is well established that polypeptides corresponding to the Cx43 CT are generated by alternate translation [6]. This mechanism includes relatively short Cx43 CT sequences, including an 11 kDa peptide that has been reported to traffic to the nucleus, with associated effects on cell-cycle progression [165]. Ongoing research might include investigation of whether there are other small, bioactive Cx43 sequences present endogenously, generated by processes such as proteolytic cleavage. Second, techniques such as cryoelectron microscopy are enabling unprecedented views of connexon structure in the context of a lipid environment at 1.9 angstrom resolution [166]. This molecular-scale insight provides an opportunity for rationale design of connexin-selective ligands, including peptidic drugs, though the disordered nature of connexin CTs may hinder implementation of this approach for therapeutic candidates targeting this domain. This is a significant challenge to surmount given, as outlined in this review, a majority of currently available therapeutic molecules mimic or target the Cx43 CT. Third, a challenge to translating the clinical promise of existing Cx43 mimetic drug candidates has been to overcome the long-standing resistance to therapeutic peptides by the pharmaceutical industry. This includes addressing matters such as the limited options for administration of this class of drug molecules, as well as the short half-life and poor tissue penetration characteristics of peptide therapeutics. Nonetheless, with the advent of delivery modalities such as exosomes, this class of connexin targeting drugs could be on the cusp of a new era in which we finally see barriers to their clinical translation significantly lowered.

## Figures and Tables

**Table 1:** Overview of Cx43 mimetic peptides and their currently known effects.

Peptide Name	Type	Sequence	Effects in Disease Models Pertinent to Heart
Gap26	Extracellular Loop	VCYDKSFPISJVR	Improves myocyte viability postischemia in vitro [46,75,76,77]
Gap27	Extracellular Loop	SRPTEKTIFII	Reduces cardiac I/R injury severity ex vivo and in vivo [77,78,79]
L2	Cytoplasmic Loop	DGVNVDMHLKQIEIKKFKYGIEEHGK	Protects myocytes against I/R injury [80]
Gap19	Cytoplasmic Loop	KQIEIKKFK	Reduces cardiac I/R injury severity in vivo [80,81,82]
αCT1	Cytoplasmic Terminus	Ant-RPRPDDLEI	Preischemic treatment decreases cardiac I/R injury severity ex vivo. In clinical testing in humans [26,83]
αCT11	Cytoplasmic Terminus	RPRPDDLEI	Reduces cardiac I/R injury ex vivo and in vivo [83,84,85]

## Data Availability

Not applicable.
