# Correction: Marsh et al. Peptidic Connexin43 Therapeutics in Cardiac Reparative Medicine. *J. Cardiovasc. Dev. Dis.* 2021, *8*, 52

**DOI:** 10.3390/jcdd9040121

**Published:** 2022-04-16

**Authors:** Spencer R. Marsh, Zachary J. Williams, Kevin J. Pridham, Robert G. Gourdie

**Affiliations:** 1 Fralin Biomedical Research Institute at VTC, Virginia Tech, Roanoke, VA 24016, USA; srmarsh@vt.edu (S.R.M.); zacharyjw@vt.edu (Z.J.W.); kjprid89@vt.edu (K.J.P.); 2 Center for Heart and Reparative Medicine Research, Virginia Tech, Roanoke, VA 24016, USA; 3 Translational Biology Medicine and Health Graduate Program, Virginia Tech, Roanoke, VA 24016, USA; 4 Department of Biomedical Engineering and Mechanics, Virginia Tech, Blacksburg, VA 24061, USA; 5 Department of Emergency Medicine, Virginia Tech Carilion School of Medicine, Virginia Tech, Roanoke, VA 24016, USA

The authors would like to make corrections to the reference citations in the original article [1], including incorrectly numbered citations and uncorrected reference items. The corrected version of this literature review addresses errors in the numbering of citations that were introduced during the proofreading stage of submission.

1. As a result of the corrections to citations, the main text needs to make the following corresponding changes:

In Table 1, “Protects myocytes against volume overload post-I/R injury” should be corrected to “Protects myocytes against”.

In Section 3.1.1, “A review characterizing the possible mechanism of action for extracellular loop peptides is found in the literature by Beyer et al.” should be corrected to “A review characterizing the possible mechanism of action for extracellular loop peptides is found in the literature by Berthoud et al.”.

In Section 3.1.2, “Other investigations of Peptide5 in preclinical models have included elucidation of its salutary effects on retinal pigment epithelial cell barrier function [107], diabetic retinopathy [108], and chronic kidney disease [109]” should be corrected to “Other investigations of Peptide5 in preclinical models have included elucidation of its salutary effects on retinal pigment epithelial cell barrier function [106] and chronic kidney disease [107]”.

2. As a result of the corrections to citations, the “References” section needs to make the following corresponding changes:

References [65–67,78,87,107,108,116,121,141,142] in the original article are deleted.

References [41–44,49–51,90,123,129,146] in the correction are newly added.

With this correction, the order of some references has been adjusted correspondingly. The Academic Editor has checked this correction. These changes do not affect the conclusions presented in the paper. The original article has been updated.
